# Decreased ATF4 expression as a mechanism of acquired resistance to long-term amino acid limitation in cancer cells

**DOI:** 10.18632/oncotarget.15828

**Published:** 2017-03-02

**Authors:** Florent Mesclon, Sarah Lambert-Langlais, Valérie Carraro, Laurent Parry, Isabelle Hainault, Céline Jousse, Anne-Catherine Maurin, Alain Bruhat, Pierre Fafournoux, Julien Averous

**Affiliations:** ^1^ Université Clermont Auvergne, INRA, UNH, Unité de Nutrition Humaine, CRNH Auvergne, F-63000 Clermont-Ferrand, France; ^2^ Department of Medical Biochemistry and Molecular Biology, CHU de Clermont-Ferrand, 63003 Clermont-Ferrand Cedex 1, France; ^3^ Institute of Cardiometabolism and Nutrition, Université Pierre et Marie Curie, INSERM, UMR S1138, Centre de Recherche des Cordeliers, 75006 Paris, France

**Keywords:** ATF4, amino acids, apoptosis, cancer, resistance

## Abstract

The uncontrolled growth of tumor can lead to the formation of area deprived in nutrients. Due to their high genetic instability, tumor cells can adapt and develop resistance to this pro-apoptotic environment. Among the resistance mechanisms, those involved in the resistance to long-term amino acid restriction are not elucidated. A long-term amino acid restriction is particularly deleterious since nine of them cannot be synthetized by the cells. In order to determine how cancer cells face a long-term amino acid deprivation, we developed a cell model selected for its capacity to resist a long-term amino acid limitation. We exerted a selection pressure on mouse embryonic fibroblast to isolate clones able to survive with low amino acid concentration. The study of several clones revealed an alteration of the eiF2α/ATF4 pathway. Compared to the parental cells, the clones exhibited a decreased expression of the transcription factor ATF4 and its target genes. Likewise, the knock-down of ATF4 in parental cells renders them resistant to amino acid deprivation. Moreover, this association between a low level of ATF4 protein and the resistance to amino acid deprivation was also observed in the cancer cell line BxPC-3. This resistance was abolished when ATF4 was overexpressed. Therefore, decreasing ATF4 expression may be one important mechanism for cancer cells to survive under prolonged amino acid deprivation.

## INTRODUCTION

Tumor cells are characterized by an uncontrolled proliferation rate. Such anarchic development of the tumor mass may be accompanied by an inadequate vascularization of the tumors [1]. As a consequence, nutrients and oxygen may not be provided sufficiently and could contribute to growth arrest and necrosis of the tumor [2]. However, tumor cells exhibit high genetic instability which can lead to the development of resistance mechanisms to nutrients and oxygen deprived environment [3]. Mechanisms concerning lack of glucose and oxygen have been extensively studied [4, 5]. In contrast, less is known on how tumor cells can adapt to the lack of lipids or amino acids. Since several years, there is an increasing interest for the role of the non-essential amino acids (glutamine, serine, glycine,..) as precursors of intermediate metabolites in the context of cancer [6, 7]. However, it is unclear how tumor cells can face a global amino acid restriction since nine essential amino acids cannot be synthesized by any mammalian cell. It remains puzzling that some tumor cells facing such a deficit are able not only to survive, but also to maintain minimal growth. In contrast, it is established that in non-tumor cells the withdrawal of one essential amino acid can promote growth arrest and ultimately apoptosis if this condition persists [8].

Two pathways are well known to be controlled by amino acid availability, GCN2 (General Control Nonderepressible 2) and mTORC1 (mammalian Target Of Rapamycin Complex 1). GNC2 is a kinase activated under amino acid starvation [9]. The activation of GCN2 induces its auto-phosphorylation that contributes to the efficient phosphorylation of its substrate, the translation initiation factor eIF2α (eukaryotic Initiation Factor 2) [10]. This event leads to a global inhibition of protein synthesis but also, derepresses the translation of specific mRNAs possessing uORF (upstream Open Reading Frame) in their 5'UTR such as the one encoding ATF4 (Activating Transcription Factor 4) [11]. Expression of this transcription factor leads to the expression of a subset of specific genes involved in the adaptation to the lack of amino acids [12], these genes being either pro-survival or pro-apoptotic. The multi-protein complex mTORC1 is a major regulator of cell growth, which coordinates anabolic and catabolic processes according to nutrients and energy environment. The activity of this complex relies on the protein mTOR which has been initially identified as the kinase targeting S6K1 (ribosomal protein S6 Kinase 1) and 4E-BP1(eIF4E-Binding Protein 1) two proteins involved in the control of protein synthesis [13].

It is established that mTORC1 activity may be induced in tumors as negative regulators of its activity are often found to be mutated in cancer cells [14]. A constant and uncontrolled activation of mTORC1 can contribute significantly to the tumorigenesis through the control of different cell functions such as translation or autophagy [15]. Moreover, the overexpression of amino acid transporters that contributes to induce mTORC1 activity is a characteristic of numerous cancer cells [16, 17]. The role of GCN2 in tumorigenesis has been studied to a lesser extent. Several studies have shown that the presence of GCN2 can contribute to tumor growth in the context of hypoxia or non-essential amino acid deprivation [18, 19]. Nevertheless, GCN2 has also been shown to contribute to cell death of cancer cells in a different setting [20]. This apparent discrepancy reflects the duality of the GCN2/eIF2α/ATF4 pathway which, as other stress pathways, can promote either survival or apoptosis [21, 22] depending on the length and intensity of the stimulus.

In order to understand how a cell can adapt to a long-term amino acid deprivation, we choose to develop a cell line able to resist to this condition from mouse embryonic fibroblasts (MEFs) rather than a tumor cell line. As resistance to amino acid deprivation mostly emerge from secondary mutations, as it is the case for resistance to treatment [23–25], using a specific tumor cell line presenting several mutations could have biased the development of our model and rendered the identification of the resistance mechanisms more difficult. Therefore, MEFs cells were cultured under selection pressure in a medium containing very low concentration of amino acids. As in Darwinian selection, few cells evolved to develop the capacity to survive in this environment, each of which grows as a clonal population. This selection allows us to generate several independent AADR (Amino Acid Deprivation Resistant) clones that were able to both survive and proliferate with low concentration of amino acids.

In these clones, the study of the two main pathways involved in the maintenance of proteostasis (i.e GCN2/eIF2α/ATF4 and mTORC1) revealed a down regulation of ATF4 expression and function. The same characteristic was also observed in a cancer cell line resistant to amino acid deprivation. Our study, in the context of a long-term amino acid deprivation, designed ATF4 not only as pro-apoptotic factor but especially as a major limiting factor in the process of acquisition of amino acid deprivation resistance.

## RESULTS

### Selection and characterization of clones able to grow in low level of amino acids

**Figure 1:**
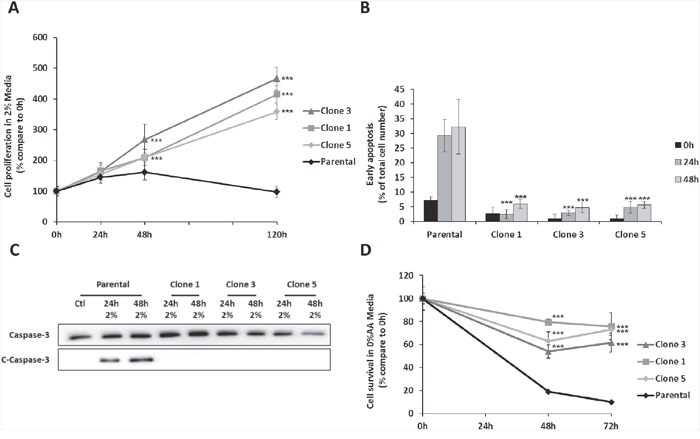
The AADR clones are resistant to the apoptosis induced by amino acid deprivation **(A)** Proliferation assay of parental cells (Parental) and 3 independent clones (Clone 1, 3, 5) cultured for 24, 48 and 120 hours in 2% medium (Concentration of amino acid compared to the control medium). Cell numbers were determined by counting. For each cell type, cell number is expressed relative to the number of cells at 0h. The graph show means ± S.E.M. of 3 independent experiments. Differences were assessed by 1-way ANOVA; *** indicates a significant difference (p<0,001) between clones and parental cells. **(B)** Early apoptosis detection using Hoechst 33342 labelling in parental cells and AADR clones cultured for 0, 24 and 48 hours in 2% medium. Results are expressed as the percentage of cells presenting high Hoechst staining. Graph shows means ± S.E.M. of 3 independent experiments. Differences were assessed by 1-way ANOVA; *** indicates a significant difference (p<0,001) between clones and parental cells. **(C)** Parental cells and AADR clones were cultured in control medium (Ctl) or in 2% medium (2%) for 24h and 48h. Immunoblot analyses of caspase 3 cleavage were performed. **(D)** Parental cells (Parental) and AADR clones (Clone 1, 2, 3) were cultured in medium lacking all amino acids for 48 hours and 72 hours. Cell numbers were determined by counting. For each cell type, cell number is expressed relative to the number of cells at 0h. Graph shows means ± S.E.M. of 3 independent experiments. Differences were assessed by 1-way ANOVA; *** indicates a significant difference (p<0,001) between parental cells and clones.

In order to generate clones able to survive and proliferate with very low level of amino acids, we progressively submitted mouse embryonic fibroblasts to a decreasing amount of amino acids. At the end of the selection process, cells were cultured in a so called “2% medium” in which the concentration of each amino acid represented 2% of the initial concentration presents in the control medium (DMEM) (see mat meth for more details). Three independent clones were selected from different culture plates for their capacity to proliferate in the 2% medium. Those clones will be further designated as Amino Acid Deprivation Resistant clones or AADR clones. When submitted to the same 2% medium, parental cells exhibit a weak proliferation during the first 48 hours (Figure 1A) together with a strong proportion of cells with a high chromatin compaction, an apoptosis marker (Figure 1B). After 48h of amino acid deficiency, the number of parental cells decreased. On the contrary, the AADR clones present a very weak percentage of apoptotic cells. This observation was corroborated by the study of the cleaved form of caspase 3. Indeed, an increase of this apoptosis marker was observed after 24 hours of amino acid limitation in the parental cells whereas no significant signal was observed in the AADR clones cultured in the same condition (Figure 1C). These results demonstrate that the AADR clones are not only able to proliferate in a low concentration of amino acids, but they have also developed the ability to resist to the apoptotic process. Strikingly, the AADR clones were also able to survive to the total absence of amino acids (Figure 1D.), a drastic situation known to induce cell death [26]. Prior to further analysis, we have checked whether the clone's ability to proliferate and survive in low level of amino acids was reversible or not. The AADR clones were cultured in complete medium for two weeks before going back to 2% medium to test whether their ability to resist to amino acid deprivation persists. We have shown (Supplementary Figure 1) that the AADR clones have kept the capacity to proliferate (with no latency) in 2% medium thus, confirming that this feature was acquired in a stable manner.

### Activity of the amino acid-regulated pathways in the AADR Clones

Amino acid availability is known to regulate mainly two pathways, i.e. the GCN2 and mTORC1 pathways. These two pathways have been characterized for their contribution to the control of proteostasis through the regulation of translation and autophagy during amino acid deprivation. We thus assessed whether the adaptation of AADR clones to amino acid deficiency resulted in a modification of one of these pathways.

**Figure 2:**
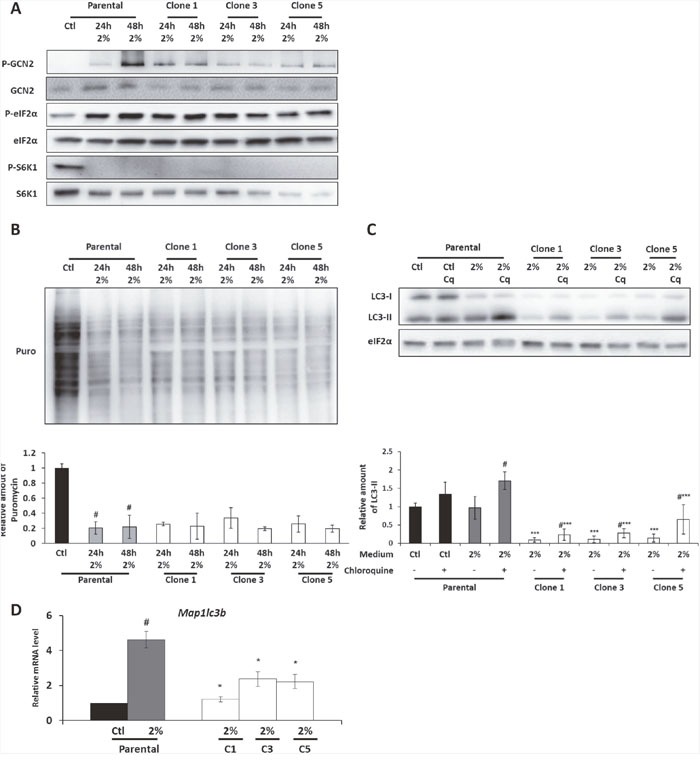
The GCN2 and the mTORC1 pathways are still regulated in the AADR clones Parental cells (Parental) and AADR clones (Clone 1, 3, 5) were cultured in control medium (Ctl) or in 2% medium (2%) for 24h and 48h **(A)** Immunoblot analyses of GCN2, eIF2α, S6K1 and their phosphorylated form were performed. **(B)** (Upper panel) Protein synthesis was measured by SUnSet method, immunoblot analyses of puromycin incorporation into proteins were performed. (Lower panel) Puromycin incorporation was determined by densitometry analysis. Graph shows means ± S.E.M. of 3 independent experiments. Differences were assessed by 1-way ANOVA; # indicates a significant difference (p<0,001) compared to parental cells in control medium. **(C)** Parental cells and AADR clones were cultured for 24h in control medium or in 2% medium. When indicated, 20μM of chloroquine (Cq) was added during the last hour of treatment. (Upper panel) Immunoblot analyses of eiF2α and LC3B processing (LC3-I and LC3-II) were performed. (Lower panel) LC3-II level was determined by densitometry analysis and normalized by red ponceau. Graph shows means ± S.E.M. of 3 independent experiments. Differences were assessed by 1-way ANOVA; *** indicates a significant difference (p<0,001) between clones and parental cells for a same treatment, # indicates a significant difference (p<0,01) between cells treated or not with chloroquine. **(D)** Parental cells and AADR clones were cultured in control medium or in 2% medium for 24h. *Map1lc3b* mRNA level was determined and normalized by the level of *β-actin* mRNA, results are expressed relative to the value observed in parental cells in control medium. Graph shows means ± S.E.M. of 5 independent experiments. Differences were assessed by 1-way ANOVA; # indicates a significant difference (p<0,001) compared to parental cells in control medium, * indicates a significant difference (p<0,05) between clones and parental cells.

In the parental cells, the 2% medium induces the phosphorylation of GCN2 and of its target eIF2α, [9] Moreover, an inhibition of S6K1 phosphorylation, a target of mTORC1 (Figure 2A) was observed in the parental cells cultured in the 2% medium. These phosphorylation states of eIF2α and S6K1 are associated with a drastic inhibition of protein synthesis in 2% medium compared to control medium as shown in Figure 2B by SUnSet technique. Likewise, a significant phosphorylation of GCN2 was also observed in the AADR clones cultured in the 2% medium (Figure 2A). We also showed that both the levels of phosphorylation of eIF2α and S6K1 and the level of protein synthesis were equivalent to those observed in the parental cells cultured in 2% medium. These results are consistent with several studies that have highlighted the importance for cells, notably tumor cells, to reduce protein synthesis level in order to face nutrient restriction [27]. It also suggests that the decrease of protein synthesis is not the limiting step concerning the ability to proliferate upon amino acid scarcity.

These two pathways are also known to regulate autophagy during amino acid deprivation [28–33]. We thus checked the level of LC3 lipidation (LC3-II, a marker of autophagy) in presence or in absence of chloroquine (an inhibitor of lysosome-autophagosome fusion), to have an insight into the autophagic flux [34]. In parental cells, chloroquine addition in 2% medium induced a strong increase of LC3-II level whereas only a faint increase was observed in control medium treated with chloroquine (Figure 2C). This demonstrates that autophagy is induced in parental cells cultured in 2% medium. As for clones, strikingly, both LC3-I and II protein levels were weaker than in the parental cells. The addition of chloroquine demonstrated that there was a significant autophagy flux in the AADR clones but apparently reduced compared to parental cells (Figure 2C).

This experiment does suggest that the difference of LC3 protein level is not due to a drastic increase in the autophagic flux but rather to a decrease of its expression. That was indeed confirmed by measuring the level of the mRNA encoding LC3 protein. A decrease of around 60% of the level of the *map1Lc3b* transcript was observed in the AADR clones cultured in 2% medium compared to parental cells (Figure 2D). Interestingly, *map1Lc3b* transcription is known to be controlled by ATF4, a downstream factor of GCN2/eIF2α pathway [35, 36].

### Expression of ATF4 and its target genes in the AADR clones

**Figure 3:**
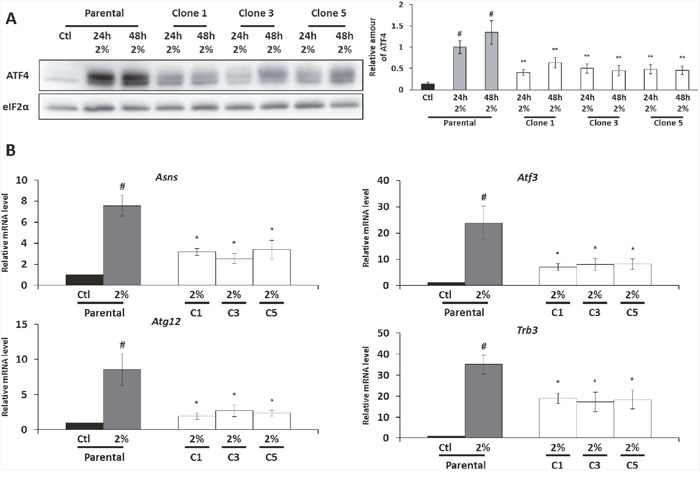
The expression of ATF4 protein and its target gene is diminished in the AADR clones Parental cells (Parental) and AADR clones (Clone 1, 2, 3) were cultured in control medium (Ctl) or in 2% medium (2%) for 24h and 48h. **(A)** (Left panel) Immunoblot analyses of ATF4 and eIF2α were performed. (Right panel) ATF4 level was determined by densitometry analysis. Graph shows means ± S.E.M. of 5 independent experiments. Differences were assessed by 1-way ANOVA. # indicates a significant difference (p<0,001) compared to parental cells in control medium., ** indicates a significant difference (p<0,01) compared to parental cells in 2% medium. **(B)** Parental cells and AADR clones were cultured in control medium or in 2% medium for 24h. *Asns*, *Atf3*, *Atg12* and *Trb3* mRNA levels were determined and normalized by the level of *β-actin* mRNA, results are expressed relative to the value observed in parental cells in control medium. Graph show means ± S.E.M. of 5 independent experiments. Differences were assessed by 1-way ANOVA; # indicates a significant difference (p<0,001) compared to parental cells in control medium, * p<0,05 compared to parental cells in 2% medium.

Considering that ATF4 is involved in the control of *map1Lc3b* transcription, we decided to investigate its protein level in the AADR clones. As expected, in parental cells, the phosphorylation of eIF2α was associated with the increase of ATF4 protein level during amino acid deprivation (Figure 3A). As previously described for a single essential amino acid deprivation [37], the level of *Atf4* mRNA was also increased by the 2% medium (Supplementary Figure 2.). Despite an equivalent level of eIF2α phosphorylation (cf. Figure 2A), the amount of ATF4 is significantly lower in the AADR clones compared to the parental cells cultivated in the 2% medium. In accordance with this result, we observed that the level of *Atf4* mRNA was also lower in the AADR clones in comparison with the parental cells (Supplementary Figure 2.). These results illustrate that AADR clones present a significant lower expression of ATF4 at both protein and mRNA level.

In order to determine whether this decrease of ATF4 expression is sufficient to impact the ATF4-dependent gene expression program, we studied, in addition to *map1Lc3b*, the mRNA expression of several ATF4-dependent genes such as *Asns, Atf3, Atg12* and *Trb3* [32, 38–40]. As expected, the increase of ATF4 expression in the parental cells cultured in 2% medium was associated with an important increase in the expression of these different genes compared to the control condition (Figure 3B). However, for all these genes, the amounts of their mRNA were significantly lower in the AADR clones compared to the parental cells cultured in 2% medium. The alteration of these ATF4-target genes regulation in AADR clones is coherent with the observation that these clones present an alteration of ATF4 expression. To rule out that the decrease in ATF4 expression and its target genes was a consequence of the long term culture in 2% medium rather than a modification linked to the adaption mechanism, we cultured the AADR clones for two weeks in the control medium before to replace them in 2% medium for 24 and 48 hours (a protocol similar to the one applied to the parental cells). This experimental paradigm shows that the down-regulation of ATF4 and its target genes is a stable trait acquires during the selection (Supplementary Figure 3).

### Role of the downregulation of ATF4 in conferring resistance to amino acid deficiency

**Figure 4:**
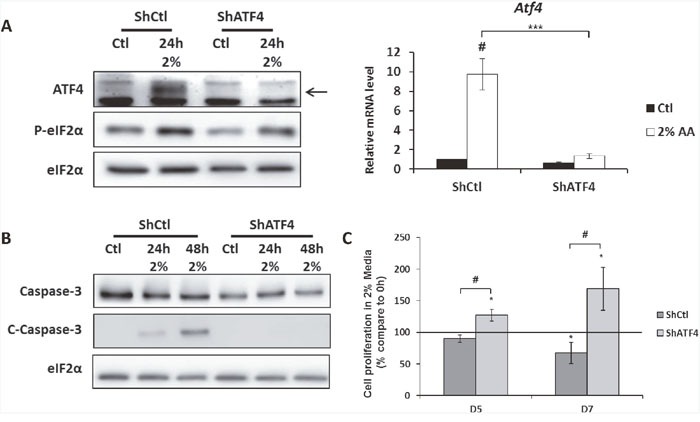
The knock-down of ATF4 expression in MEFs increases cell survival during amino acid deprivation MEFs were infected with a lentivirus expressing either a ShRNA control (ShCtl) or a ShRNA targeting ATF4 (ShATF4). **(A)** ShCtl cells (ShCtl) and ShATF4 cells (ShATF4) were cultured in control medium (Ctl) or in 2% medium (2%) for 24 hours. (Left panel) Immunoblot analyses of ATF4 and eIF2α were performed. (Right panel) ATF4 mRNA level was determined and normalized by the level of *β-actin* mRNA, results are expressed relative to the value observed in ShCtl cells in control medium. Graph shows means ± S.E.M. of 3 independent experiments. Differences were assessed by 1-way ANOVA; # indicates a significant difference (p<0,001) compared to control medium *** indicates a significant difference (p<0,001) compared to ShCtl cells in 2% medium. **(B)** ShCtl cells and ShATF4 cells were cultured in control medium or in 2%medium for 24 and 48 hours. Immunoblot analyses of caspase 3 cleavage were performed. **(C)** ShCtl cells and ShATF4 cells were cultured in 2% medium for 5 days (D5) and 7 days (D7). Cell numbers were determined by counting. For each cell type, cell number is expressed relative to the number of cells at 0h (represented by the black line). Graph shows means ± S.E.M. of 3 independent experiments. Differences were assessed by 1-way ANOVA; # indicates a significant difference (p<0,01) compared to ShCtl cells, ** indicates a significant difference (p<0,01) compared to 0h.

It was crucial to determine whether this down-regulation of ATF4, acquired during the selection, contributes to the adaption to the low amino acid environment or if it is just a consequence of this adaption. To assess this question, MEFs cells were infected with a lentivirus containing a shRNA targeting *Atf4* mRNA or a control shRNA. As expected, the level of *Atf4* mRNA and protein were dramatically decreased in the cells infected with the shRNA ATF4 cultivated in the 2% medium compared to the cells infected with the shRNA control (Figure 4A). This decrease of ATF4 expression resulted in the decrease of *Asns* mRNA level, confirming the functional impact of ATF4 knock-down (Supplementary Figure 4). Then, the consequence of the down regulation of ATF4 expression on cell survival in 2% medium was determined. Knocking-down ATF4 in parental cells resulted in an absence of caspase 3 cleavage after 24h and 48h in 2% medium whereas caspase 3 cleavage is observed in parental cells infected with the control shRNA (Figure 4B). As for proliferation, the absence of ATF4 consecutive to the infection with Atf4-shRNA enables control cells to slightly proliferate after 5 and 7 days in 2% medium whereas the number of cells significantly decreases after infection with the control shRNA (Figure 4C). Reciprocally we tested if the overexpression of ATF4 in the AADR clone would decrease their resistance to amino acid limitation. The overexpression obtained by infecting the cells with an adenovirus expressing ATF4 led to a very variable level of expression of ATF4 between the different clones (Supplementary Figure 5), this feature represents a limit for the interpretation of the data. Nevertheless, for each clones the increase of ATF4 expression was associated with the cleavage of the caspase 3 and an arrest of cell growth. Together, these results demonstrate that the decrease of ATF4 expression increases the ability of cells to survive during prolonged amino acid deprivation.

### ATF4 expression in amino acid deficiency-resistant cancer cell lines

It is already admitted that ATF4 can contribute to cell death during prolonged or intense stress [21]. Our knock-down experiments confirm this concept for MEFs in the setting of a partial amino acid starvation. We made the hypothesis that a similar adaptation to amino acid deprivation involving a low level of expression of ATF4 could be observed in a cancer cell line. We choose to focus our study on pancreatic cancer cell lines. Indeed, pancreatic tumors present a high metabolism and a dense stroma which conduct to an impaired development of the tumor vasculature and thus, to the development of hypoxic and nutrient deprived region [41, 42].

**Figure 5:**
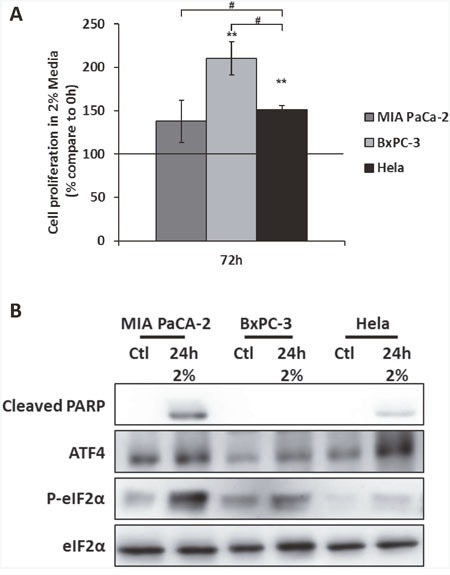
The expression of ATF4 is associated to the resistance to amino acid deprivation in the cancer cell line BxPC-3 **(A)** HeLa, MIA PaCa-2 and BxPC-3 were cultured in 2% medium (2%) for 72 hours. Cell numbers were determined by counting. For each cell type, cell number is expressed relative to the number of cells at 0h (represented by the black line). Graph shows means ± S.E.M. of 3 independent experiments. Differences were assessed by 1-way ANOVA; # indicates a significant difference (p<0,001) between cell lines, ** indicates a significant difference (p<0,01) compared to 0h. **(B)** MIA PaCa-2, BxPC-3 and HeLa cells were cultured control medium or 2% medium for 24 hours. Immunoblot analyses of ATF4, eIF2α and its phosphorylated form and the cleaved form of PARP were performed.

We tested two human pancreatic cancer cell lines: MIA PaCa-2 and BxPC-3. MIA PaCa-2 cells have been characterized for their poor tolerance to nutrient deprivation whereas BxPC-3 have been shown to be more resistant [43]. Based on our previous study, we also used HeLa cells, a cervical cancer cell line, as a positive control of ATF4 expression under amino acid deprivation [37]. Among those cell lines, BxPC-3 cells exhibited the strongest increase of their cell number after 3 days of culture in 2% medium (Figure 5A). In addition, relatively to the cell number observed after 3 days in control medium, the BxPC3 cell line exhibited a decrease of only 40% in 2% medium (Supplementary Figure 6). Comparatively, HeLa and MIA PaCa-2 presented a strong decrease, respectively of 90% and 95% of their cell numbers. In these two cell lines, the strong inhibition of cell growth was accompanied by the appearance of the cleaved form of PARP, a target of caspase-3 (Figure 5C). In BxPC-3, the cleaved form of PARP was not detected, suggesting that the 2% medium did not induce apoptosis in this cell line. Remarkably, this capacity of the BxPC-3 to resist to apoptosis was associated with the weaker level of expression of ATF4 detected among the three cell lines tested (Figure 5B).

**Figure 6:**
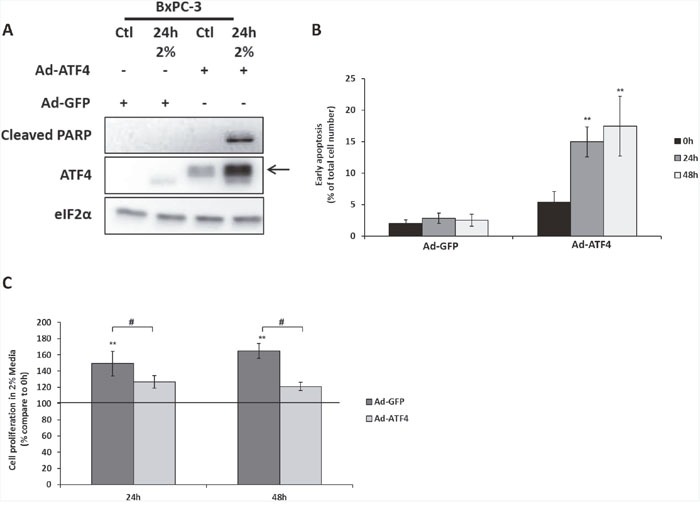
The overexpression of ATF4 in BXPC-3 induces cell death and reduces cell proliferation during amino acid deprivation BxPC-3 cells were infected with adenovirus expressing either GFP (Ad-GFP) or mouse form of ATF4 (Ad-ATF4). **(A)** Cells infected with either Ad-GFP or Ad-ATF4 were cultured in control medium (Ctl) or in 2% medium (2%) for 24 hours. Immunoblot analyses of ATF4, eIF2α and the cleaved form of PARP were performed. The arrow indicates the mouse form of ATF4. **(B)** Early apoptosis detection using hoechst 33342 labelling in cells infected with either Ad-GFP or Ad-ATF4 cultured in 2% medium for 24 and 48 hours. Results are expressed as the percentage of cells presenting high hoechst staining. Graph shows means ± S.E.M. of 3 independent experiments. Differences were assessed by 1-way ANOVA; ** indicates a significant difference (p<0,01) between compared to 0h. **(C)** Cells infected with either Ad-GFP or Ad-ATF4 were cultured in 2% medium for 24 and 48 hours. Cell numbers were determined by counting. For each cell type, cell number is expressed relative to the number of cells at 0h (represented by the black line). Graph shows means ± S.E.M. of 3 independent experiments. Differences were assessed by 1-way ANOVA; # indicates a significant difference (p<0,001) between cells infected with Ad-GFP and Ad-ATF4, ** indicates a significant difference (p<0,01) compared to 0h.

Thus, the capacity of a human tumor cell line to survive in low amino acid environment could be due to a decreased expression of ATF4. Therefore, overexpressing ATF4 in BxPC3 cells may be a good strategy to sensitize them to the 2% medium. Indeed, overexpression of ATF4 by adenovirus infection leads to the induction of apoptosis of BxPC-3 cells in 2% medium as shown by the detection of the cleaved form of PARP (Figure 6A) and the significant increase of cells with a high chromatin compaction (Figure 6B). Moreover, this apoptosis induction was associated with a reduction of proliferation compared to BxPC-3 cells infected with Ad-GFP (Figure 6C). All together, these results confirm the hypothesis that a weak level of expression of ATF4 can contribute to the capacity of a subset of tumor cells to survive and proliferate in low concentration of amino acids.

## DISCUSSION

**Figure 7:**
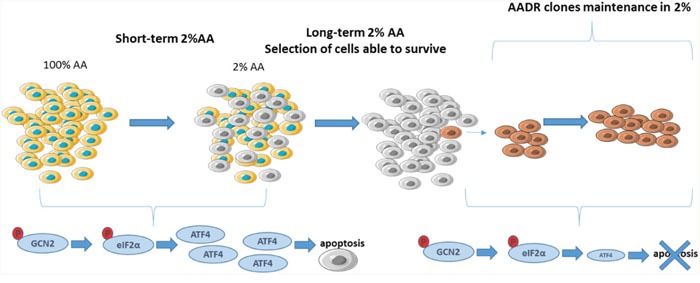
Summary diagram of the AADR clones In the short term, the 2% amino acid medium induces rapidly the apoptosis of mouse embryonic fibroblasts, through notably the induction of ATF4 expression. Nevertheless, this high selective pressure results in the selection of Amino Acid Deprivation Resistant (AADR) clones. These clones present an alteration of the activation of the GCN2/eIF2α/ATF4 pathway, despite the phosphorylation of GCN2 and eIF2α, the level of ATF4 remains low, preventing them from apoptosis.

The aim of this study was to identify mechanisms of resistance that can occur in tumor cells due to the pressure of selection from an amino acid-deprived environment. For that purpose, we generated clones able to grow in a medium containing low amount of amino acids (Figure 7.). It is noticeable that parental cells exposed to a very low level of amino acids exhibit after 24 hours the initiation of apoptosis while their growth is not yet totally abolished. In contrast, the AADR clones were resistant to the apoptosis induced by a very low concentration of amino acids.

Until now two signaling pathways, mTORC1 and GCN2/eIF2α/ATF4, are well known to be involved in the adaption to the variation of amino acid supply. The mTORC1 pathway did not appear to behave differently in the AADR clones and the parental cells since in both cases S6K1 phosphorylation was abolished upon amino acid limitation. If the inhibition of mTORC1 has been proposed as a therapeutic strategy to treat cancers, it is now established that its inhibition alone may not be sufficient to induce apoptosis [44].

Interestingly, we observed an alteration of the eIF2α/ATF4 pathway in the AADR clones. Indeed, compared to the parental cells, the level of ATF4 protein was decreased in the clones despite an equivalent level of eIF2α phosphorylation. It remains complex to determine the cause of this low expression level as ATF4 expression is highly regulated at the level of its transcription, translation and protein stability [45]. The low amount of ATF4 protein is associated with a decreased amount of its mRNA. But, it cannot be concluded that the decrease of *Atf4* mRNA is the initial event contributing to a low ATF4 protein level as it has been suggested that ATF4 might activate its own transcription [46]. ATF4 is well known to be highly regulated at the translational level [47]. Nevertheless, the levels of both eIF2α phosphorylation and protein synthesis observed in the AADR clones suggest that the under-expression of ATF4 is not due to a defect of its translation. ATF4 expression is also tightly regulated at the level of protein degradation by the proteasome [45]. This regulation involved the ubiquitin ligase βTrCP, this enzyme targets notably tumor suppressor proteins for proteasomal degradation [48]. It will be interesting to determine whether this process could be involved in the low level of ATF4 expression in the AADR clones. If further studies would be necessary to identify the exact cause of this under expression, it has to be kept in mind that it may differ for each AADR clone.

The fact that the down regulation of ATF4 and its target genes are common characteristics of the tested independent clones suggests that this alteration may have an important role in the resistance to amino acid deprivation. This concept has been confirmed by the knock-down of ATF4 in the parental cells which have acquired a resistance to the apoptosis induced by amino acid deprivation. This pro-apoptotic role of ATF4 is in accordance with data of the literature showing that the induction of ATF4 expression, by drugs or glutamine deprivation [49, 50], has a pivotal role in the initiation of apoptosis [51].

However, other studies have demonstrated that ATF4 is necessary for the survival of tumor cells in deprived environment [18]. ATF4 has been shown to be necessary for the regulation of VEGF expression in low vascularized area to promote angiogenesis and so, the furniture of amino acids [18]. The study of Ye *et al*. demonstrates that the presence of ATF4 is necessary to synthesize non-essential amino acids and more particularly asparagine [19]. Nevertheless, the authors used for their *in vitro* experiments a medium lacking the non-essential amino acids. Our model differs from that approach as the scarcity concerns all the amino acids and in particular the essential ones that cannot be synthetized. These two examples illustrate the complexity to establish a model concerning the role of ATF4 in the survival/apoptosis balance in different contexts of amino acid deprivation. This complexity resides in the fact that ATF4 increases the expression of numerous pro-survival and pro-apoptotic genes. In addition, the capacity to resist to amino acid scarcity is likely to reside in the modification of expression of a set of genes rather than in the down regulation of only one pro-apoptotic gene such as *Trb3* or *Atf3*. Our data clearly show that ATF4 down regulation protects from the apoptosis induced by the long-term amino acid deprivation.

It is reasonable to make the hypothesis that other mechanisms, independently of ATF4 down regulation, could contribute to the resistance of the AADR clones. Another important observation is that the phosphorylation of eIF2α is conserved in the AADR clones, suggesting that this event could be necessary to face the amino acid deprivation. This hypothesis is in accordance with the study of Han *et al*. which has demonstrated that the phosphorylation of eIF2α and the resulting inhibition of protein synthesis are in favor of survival [52]. The importance of protein synthesis limitation to resist to long term amino deprivation is well illustrated by the study of Leprivier *et al* [27]. The authors demonstrate that the decrease of the elongation rate through the activation of the eukaryotic Elongation Factor 2 Kinase (eEF2k) promotes cell survival during nutrient deprivation. Moreover, a high level of eEF2K is associated with poor diagnostic in different kind of tumors [27]. The protective effect of eEF2K has been shown to rely on the inhibition of protein synthesis and not on the induction of autophagy [53] which has been reported to be induced by eEF2K [54, 55]. Similarly, in the AADR clones our results suggest that their capacity to survive in amino acid-deprived medium do not rely on the increase of autophagy. Indeed, the levels of autophagic genes were decreased and the total autophagic flux seems to be decreased. As already mentioned, a high level of autophagy can represent only a transient adaption to a lack of amino acids. Moreover, it has been demonstrated that a link exists between autophagy and the induction of apoptosis [8], consequently, limiting the autophagy may represent an efficient strategy to resist to a long term amino acid deprivation.

Finally, we have been able to establish that this feature of a low level of ATF4 expression leading to amino acid deprivation resistance occurred also in, at least, one cancer cell line. Indeed, BxPC-3 pancreatic cancer cell line exhibited both the capacity to grow in amino acid-deprived medium and a low level of ATF4 expression. Moreover, overexpression of ATF4 in BxPC-3 cells re-sensitizes them to the apoptosis induced by amino acid deprivation. As cancer cells emerged from a primary mutation which could be followed by others, it remains striking to observe the same characteristics of amino acid deprivation resistance in BxPC-3 and in the AADR clones. Despite the presence of differential selection pressures and maybe different mechanisms between AADR clones and BxPC-3, the decreased expression of ATF4 seems to be an important characteristic in order for cell to survive to amino acid deprivation. This observation establishes that the cellular model that we developed is pertinent to identify a feature of the resistance to amino acid deprivation that can be shared by cancer cells. Importantly, our study does not aim at demonstrating a strict correlation between ATF4 expression and cell survival upon amino acid deprivation. As mentioned above others mechanisms of resistance may arise in tumor cells. Yet, increasing the level of expression of ATF4 might be an efficient strategy to target specific tumor cells able to resist to deprived environment, not only because ATF4 can be a pro-apoptotic factor but also, because low level of ATF4 might be the cause of the resistance. For that reason, the comprehension of the causes of the decrease in ATF4 expression would be of particular importance. It remains that strategies to target ATF4 expression will have to be considered in the complexity of tumor heterogeneity and it is likely that combined or sequential therapies will be necessary.

## MATERIALS AND METHODS

### Cell culture and treatment conditions

Mouse embryonic fibroblasts (MEFs) were cultured at 37°C in humidified 5% CO2 atmosphere in control medium prepared from Dulbecco's Modified Eagle's Medium (Sigma) containing 10% fetal bovine serum and supplemented with non-essential amino acid mix (Gibco), glutamine, penicillin/streptomycin and gentamycin. HeLa cervical cancer cell line, MIA PaCA-2 and BxPC-3 pancreatic cancer cell lines were cultured in control medium. MIA PaCA-2 and BxPC-3 were kindly given by Dr. S. Pyronnet (CRCT, INSERM, Toulouse, France). Prior to amino acid starvation experiments, cells were washed twice with phosphate-buffered saline (PBS) and refed with the appropriate medium.

To obtain cells resistant to amino acid deprivation, MEFs cells were seeded in several dish plates and exposed to mediums with decreased concentration of amino acids. Those mediums were made by diluting the control medium with DMEM lacking all amino acids and supplemented with 10% of dialyzed fetal bovine serum. The concentration of amino acids was gradually decreased during several weeks causing severe growth arrest and cell death. The last remaining cells were exposed to the medium containing only 2% of the amino acids present in the control medium (medium named 2% medium) again for several weeks. Few cells survived and formed individual clones. Three clones were selected form 3 independent plates and were continuously cultured in the 2% medium. The cells from which clones were derived were thus called parental cells.

### Analysis of gene expression using real time RT-PCR

Total RNA was prepared using RNeasy mini kit (Qiagen) and treated with DNase I, Amp Grade (Invitrogen, Carlsbad, CA, USA) prior to cDNA synthesis. RNA integrity was electrophoretically verified by ethidium bromide staining. RNA (1 μg) was reverse transcribed with 100 U of Superscript II plus RNase H^−^ Reverse Transcriptase (Invitrogen) using 100 μM random hexamer primers (Amersham Biosciences, Piscataway, NJ, USA), according to the manufacturer's instructions. Real-time quantitative PCR was carried out on a Bio-Rad CFX-96 detection system with quantitative qPCR SYBR Green reagents (Bio-Rad, Hercules, CA, USA) and with a primer concentration of 0.5 μM. For a list of primer sequences, see Supplementary Table 1. PCR conditions were standardized to 39 cycles of: 95°C for 08 s, 59°C for 05 s and 72°C for 10 s.

### Antibodies

eIF2α, P-eIF2α (Ser51), Total S6K1, P-S6K1 (T389), Cleaved PARP, total and cleaved caspase-3 antibodies were purchased from Cell Signaling Technologies, ATF4 (SC-200) from Santa Cruz and MAP1LC3B antibody from Novus. Antibody Puromycin (clone 12D10) was kindly given by Phillipe Pierre (Centre d'Immunologie de Marseille-Luminy, France).

### Immunoblot analysis

Cells were lysed in a lysis buffer containing (50 mM Tris, 25 mM β-glycerophosphate, 50 mM NaCl, 1 mM EDTA, 1 mM EGTA, 0.5% (v/v) Triton X100, 1 mM DTT, 1 mM benzamidine, protease and phosphatase inhibitor cocktail (Sigma). Proteins were separated by SDS-polyacrylamide gel electrophoresis and transferred onto a Hybond-P PVDF membrane (Amersham Biosciences). Membranes were blocked for 1 h at room temperature with a solution of 5% nonfat milk powder in TN (50mM Tris-HCL, pH 8.0, 150mM NaCl, 0.1% Tween-20). The blots were then incubated with antibody in blocking solution overnight at 4°C. Antibodies were diluted according to the manufacturer's instructions. The blots were washed three times in TN and incubated with horseradish peroxidase conjugated goat anti-IgG (1:5000) (Santa Cruz, CA) in blocking buffer for 1 h at room temperature. After three washes, the blots were developed using the LuminataTM Western HRP substrate (Millipore Billerica, MA).

### Establishment of stable cell lines expressing ShRNA

The lentiviral vectors harboring ATF4 and control shRNA sequences were obtained from Sigma-Aldrich (Mission TRC shRNA collections). The lentiviral particles were produced by EVIR (Enveloppes Virales et Ingénierie des Rétrovirus, Unité de Virologie Humaine, Inserm U758, Ecole Normale Supérieure de Lyon, France). MEFs cells were seeded in 6 wells plates and infected the next day with lentivirus expressing either a control ShRNA or the ShRNA targeting ATF4 at a Multiplicity of Infection (MOI) of 25. Twenty four hours after the infection, cells were washed twice and refed with DMEM medium containing the selective agent puromycin.

### Hoechst 33342 staining assay/Fluorescent microscopy evaluation of cell apoptosis and morphology

Cells were stained with 2 μg/mL of Hoechst 33342 for 5 min at 37°C. Then, cells were washed twice with PBS and refed with amino acid-deprived medium. Hoechst 33342 fluorescence was assessed using Zen microscope (Zeiss) with DAPI filter (Excitation 353 nm/Emission 465 nm). Early apoptotic cells were detected by strong Hoechst staining which represented the condensation of nuclear chromatin and its fragmentation. For each cell types and conditions, several random areas were selected for a total count of approximatively 300 cells. The number of apoptotic cells was normalized to the total number of cells in each area.

### Cell proliferation assays

Cells were plated in 12-wells plates and, the next day, cultured in either control medium or in amino acid-deprived medium. The proliferation rate of each cell types was assessed by counting 3 wells for each time points using Beckman coulter counter Z1. The number of cells was normalized according to the number of cells counted at the beginning of the experiment.

### Adenoviral infection

Cells were plated the day prior infection. The next day, cells were washed twice and refed with control medium without FBS. Infection with adenovirus expressing either GFP or mouse ATF4 was carried at a multiplicity of infection of 100. After 8h of infection, FBS was added to the medium for a final concentration of 10%. After 24h of infection, the cells were washed twice and refed with either control medium or 2% medium. Adenovirus were kindly given by Guozhi Xiao (Rush University Medical Center, Chicago, USA).

### Measure of protein synthesis level by SUnSet method

The level of protein synthesis has been determined by the SUnSet method [56]. Briefly, during the last 30 min of treatment puromycin (Sigma-Aldrich) at 5μg/ml was added to the culture medium. Cells were harvested and lysed for immunoblot analysis with anti puromycin antibody.

### Statistical analysis

All data are expressed as means ± SEM. Differences between samples were assessed by one-way ANOVA (fisher test).

## SUPPLEMENTARY FIGURES AND TABLE


