# Supplementary material for: Decreased ATF4 expression as a mechanism of acquired resistance to long-term amino acid limitation in cancer cells
Source: Oncotarget. 2017 Mar 2;8(16):27440–53. doi: 10.18632/oncotarget.15828 (PMC5432347; doi:10.18632/oncotarget.15828)
Supplement: Supplementary file 1 [file oncotarget-08-27440-s001.pdf]

Decreased ATF4 expression as a mechanism of acquired resistance to long-term amino acid limitation in cancer cells

SUPPLEMENTARY MATERIALS

SUPPLEMENTARY FIGURES AND TABLE

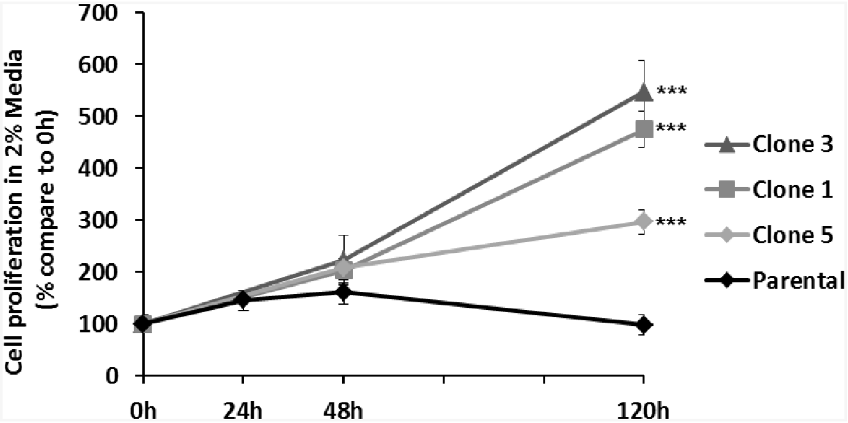

Supplementary Figure 1: The capacity of the AADR clones to grow in 2% medium is a stable characteristic. AADR clones (Clone 1, 3, 5) were cultured for 1 week in control medium. Then, parental cells (Parental) and AADR clones were cultured in 2% medium for 24, 48 and 120 hours. Cell numbers were determined by counting. For each cell type, cell number is expressed relative to the number of cells at 0h. Graph shows means ± S.E.M. of 3 independent experiments. Differences were assessed by 1-way ANOVA; *** indicates a significant difference (p<0,001) between parental cells and clones.

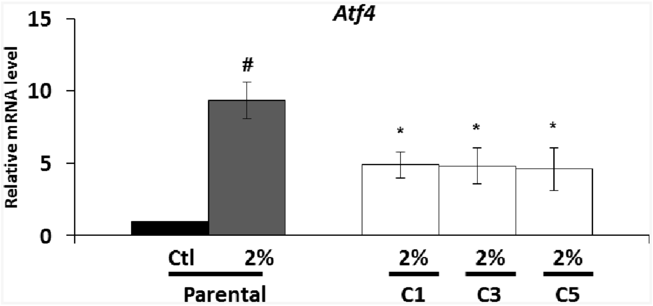

Supplementary Figure 2: The expression of ATF4 mRNA is decreased in the AADR clones. Parental cells (Parental) and AADR clones (C1, C3, C5) were cultured in control medium (Ctl) or in 2% medium (2%) for 24 hours. *Atf4* mRNA level was determined and normalized by the level of *β-actin* mRNA, results are expressed relative to the value observed in parental cells in control medium. Graph shows means ± S.E.M. of 5 independent experiments. Differences were assessed by 1-way ANOVA; # indicates a significant difference (p<0,001) compared to parental cells in Ctl medium for 24h, * indicates a significant difference (p<0,05) compared to parental cells in 2% medium for 24h.

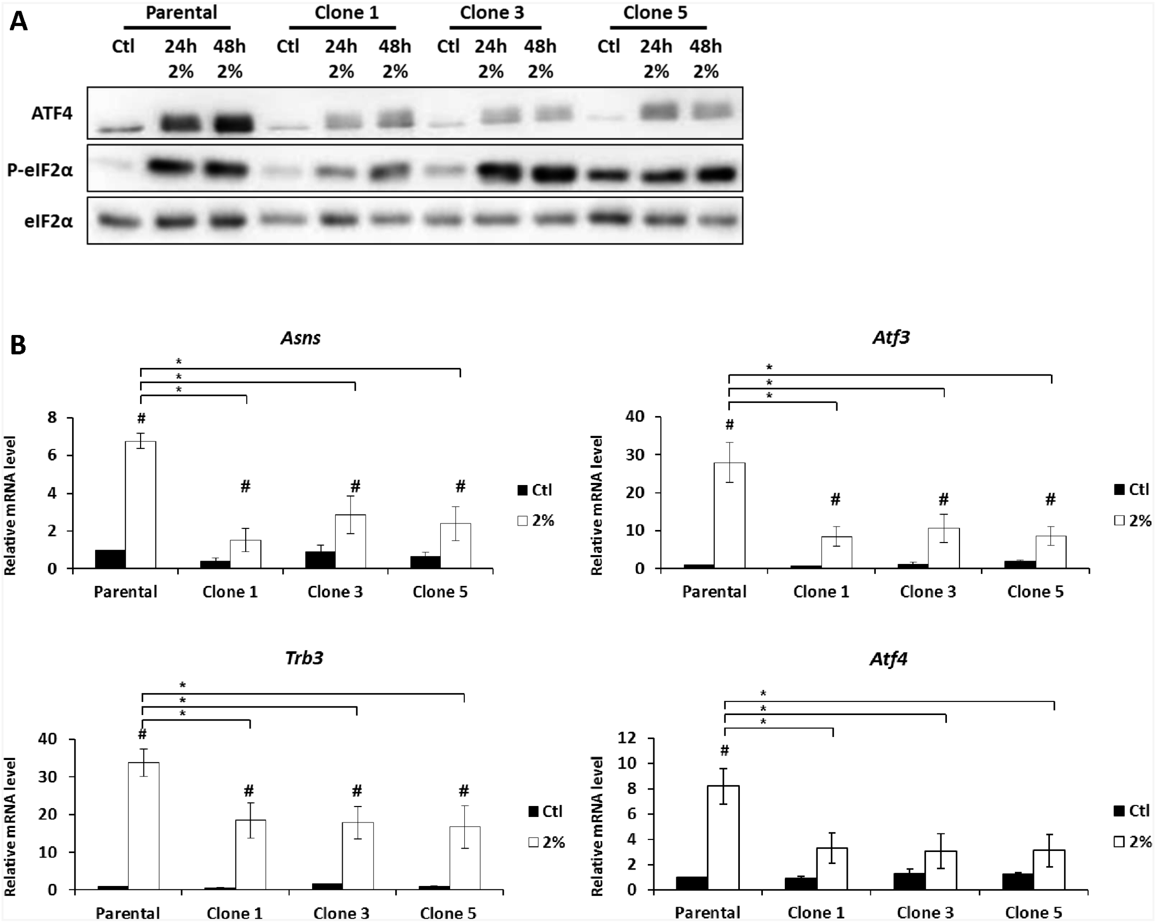

Supplementary Figure 3: The decrease of ATF4 protein level is a stable characteristic of the AADR clones. AADR clones (Clone 1, 3, 5) were cultured for 2 weeks in control medium prior to the experiment. **(A)** Parental cells (Parental) and AADR clones (Clone 1, 2, 3) were cultured in control medium (Ctl) or in 2% medium (2%) for 24 hours and 48 hours. Immunoblot analysis of ATF4, eIF2α and its phosphorylated form were performed. **(B)** Parental cells and AADR clones were cultured in control medium and in 2% medium for 24 hours. *Asns*, *Atf3*, *Atg12* and *Trb3* mRNA levels were determined and normalized by the level of *β-actin* mRNA, results are expressed relative to the value observed in parental cells in control medium. Graph show means ± S.E.M. of 5 independent experiments. Differences were assessed by 1-way ANOVA; # indicates a significant difference (p<0,01) compared to control medium, * indicates a significant difference (p<0,05) compared to parental cells in 2% medium.

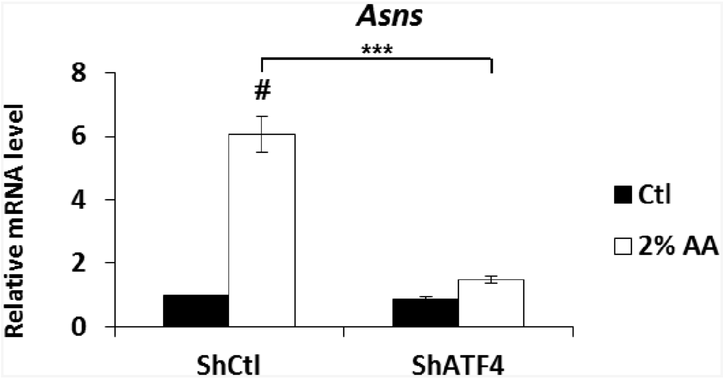

Supplementary Figure 4: The expression of *Asns* mRNA is decreased in cells expressing ShATF4. MEFs were infected with a lentivirus expressing either a ShRNA control (ShCtl) or a ShRNA targeting ATF4 (ShATF4). **(A)** ShCtl cells (ShCtl) and ShATF4 cells (ShATF4) were cultured in control medium (Ctl) or in 2% medium (2%) for 24 hours. *Asns* mRNA levels were determined and normalized by the level of *β-actin* mRNA, results are expressed relative to the value observed in parental cells in control medium. Graph shows means ± S.E.M. of 3 independent experiments. Differences were assessed by 1-way ANOVA; # indicates a significant difference (p<0,001) compared to control medium, *** indicates a significant difference (p<0,001) between ShCtl and ShATF4 cells in 2% medium.

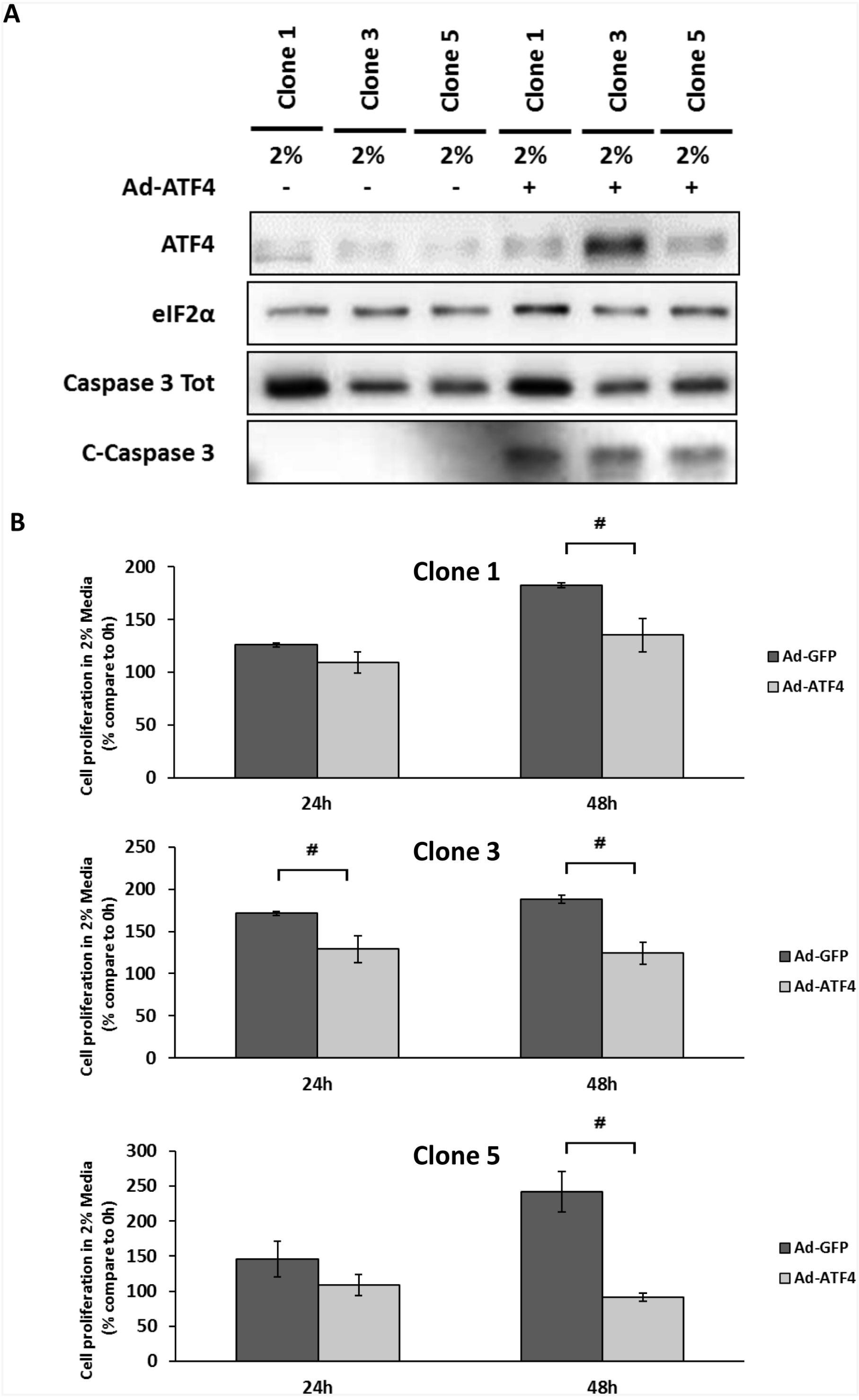

Supplementary Figure 5: The overexpression of ATF4 in the clones AADR induces the cleavage of the caspase 3. AADR clones (Clone 1, 3, 5) were infected with adenovirus expressing either GFP (Ad-GFP) or mouse form of ATF4 (Ad-ATF4). **(A)** Cells infected with either Ad-GFP or Ad-ATF4 were cultured in 2% medium (2%) for 24 hours. Immunoblot analyses of ATF4, eIF2α, Caspase 3 and the cleaved form of Caspase 3 were performed. **(B)** Cells infected with either Ad-GFP or Ad-ATF4 were cultured in 2% medium (2%) for 24 hours and 48 hours. Cell numbers were determined by counting. For each cell type, cell number is expressed relative to the number of cells at 0h. Graph shows means ± S.E.M. of 3 independent experiments. Differences were assessed by 1-way ANOVA; # indicates a significant difference (p<0,05) between Ad-GFP and Ad-ATF4 cells.

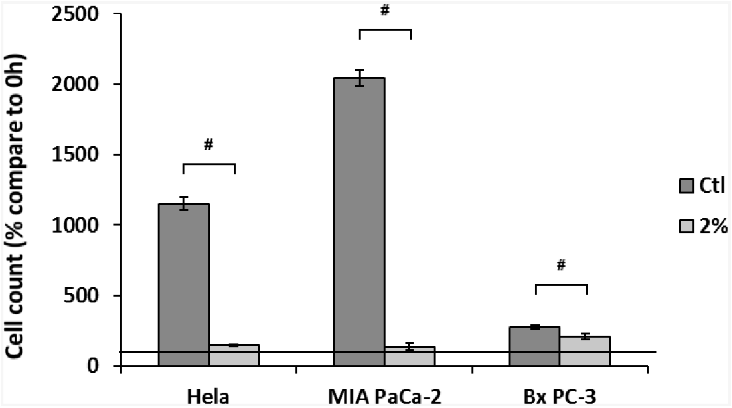

Supplementary Figure 6: Cancer cell line proliferation in Ctl and 2% medium. HeLa, MIA PaCa-2 and BxPC-3 were cultured in Control (Ctl) and 2% medium (2%) for 72 hours. Cell numbers were determined by counting. For each cell type, cell number is expressed relative to the number of cells at 0h (represented by the black line). Graph shows means ± S.E.M. of 3 independent experiments. Differences were assessed by 1-way ANOVA; # indicates a significant difference ($p<0,05$) between Ctl and 2% conditions.

Supplementary Table 1: qPCR primers

Gene	Forward	Reverse
Asns	5’-TACAACCACAAGGCGCTACA-3’	5’-AAGGGCCTGACTCCATAGGT-3’
Atf3	5'-CCAGGTCTCTGCCTCAGAAG-3'	5'-CATCTCCAGGGGTCTGTTGT-3'
Atf4	5’-TCGATGCTCTGTTTCGAATG-3’	5’-AGAATGTAAAGGGGGCAACC-3’
Atg12	5’-GGAGACACTCCTATAATGAAA-3’	5’-ATAAATAAACAACTGTTCCGA-3’
Map1lc3b	5’-CGTCCTGGACAAGACCAAGT-3’	5’-ACCATGTACAGGAAGCCGTC-3’
Trb3	5'-CCAGAGATACTCAGCTCCCG-3'	5'-GAGGAGACAGCGGATCAGAC-3'
β-actin	5′-TACAGCTTCACCACCACAGC-3′	5′-AAGGAAGGCTGGAAAAGAGC-3′
